# In silico and in vitro assessment of antimicrobial activity of *Arctium lappa L.* leaf and flower essential oil against WHO priority pathogens

**DOI:** 10.1038/s41598-026-48731-9

**Published:** 2026-04-16

**Authors:** Sawsen Rebhi, Calvin R. Wei, Najla Sadfi-Zouaoui, Abdelmonaem Messaoudi

**Affiliations:** 1 Laboratoire de Mycologie Pathologies et Biomarqueurs (LR16ES05), Université de Tunis-El Manar, Tunis, 2092 Tunisia; 2 Department of Research and Development Shing Huei Group , Taipei, Taiwan; 3 Faculty of Sciences , Gabès University , Zrig, Gabès, 6072 Tunisia

**Keywords:** *Arctium lappa*, Essential oil, Antimicrobial resistance, WHO priority pathogens, DNA gyrase, CYP51, Ethnopharmacology, Biochemistry, Biotechnology, Computational biology and bioinformatics, Drug discovery, Microbiology, Plant sciences

## Abstract

Antimicrobial resistance (AMR) poses an escalating global threat, demanding the discovery of innovative therapeutics with novel mechanisms of action. Essential oils (EOs) from medicinal plants are promising candidates due to their multitarget effects and reduced risk of resistance development. This study investigates the chemical profile and antimicrobial mechanisms of essential oil obtained from the aerial parts (leaves and flowers) of *Arctium lappa* (ALEO), a species with strong ethnopharmacological relevance yet poorly characterized in Tunisia. Gas chromatography–mass spectrometry (GC–MS) revealed 1,3-cyclooctadiene (48.6%), caryophyllene oxide (31.7%), and aromadendrene (12.0%) as major constituents. ALEO demonstrated potent in vitro antimicrobial activity against methicillin-resistant *Staphylococcus aureus* (MRSA ATCC 43300), *Pseudomonas aeruginosa* ATCC 27,853, and *Candida auris* clinical isolates, with MIC values comparable to reference drugs. Complementary molecular docking identified strong binding affinities of caryophyllene oxide and aromadendrene to bacterial DNA gyrase B and fungal lanosterol 14α-demethylase, while molecular dynamics simulations confirmed stable ligand–protein interactions (RMSD < 2.5 Å) and favorable binding energies (ΔG ≤ − 17 kcal/mol). Together, these findings highlight ALEO as a multitarget antimicrobial agent with therapeutic potential against WHO-priority pathogens, bridging traditional medicinal knowledge and modern drug discovery approaches.

## Introduction

The relentless rise of antimicrobial resistance (AMR) represents one of the gravest global health challenges, threatening decades of medical progress. If unmitigated, AMR is projected to cause up to 10 million deaths annually by 2050, surpassing cancer as the leading cause of mortality worldwide^1^. This threat is further exacerbated by a stagnating antibiotic discovery pipeline: only a small fraction of clinical candidates belong to truly novel classes or possess unique mechanisms of action^2^. The diminishing efficacy of current antimicrobials against multidrug-resistant pathogens—including WHO critical-priority species such as *Pseudomonas aeruginosa* and *Candida auris*^3^—underscores the urgent need for innovative agents with distinct pharmacological profiles capable of circumventing established resistance mechanisms. Pathogens develop antimicrobial resistance through mechanisms such as enzymatic drug inactivation, target modification, efflux pump overexpression, reduced cell permeability, and horizontal gene transfer, which collectively contribute to multidrug resistance in WHO-priority pathogens^4^.

Natural products, particularly those derived from aromatic and medicinal plants (AMPs), offer a rich source of structurally diverse bioactive compounds that may overcome conventional resistance pathways. Tunisia, located in the Mediterranean basin, harbors exceptional plant biodiversity due to its varied climatic and pedoclimatic zones^5^. Its flora encompasses both cultivated species (e.g., chamomile, thyme, eucalyptus) and extensive wild populations covering over 400,000 hectares (e.g., *Rosmarinus officinalis*, *Myrtus communis*, *Arctium lappa*)^6^. Essential oils (EOs) derived from these plants are especially promising, as their complex phytochemical composition often confers multitarget antimicrobial effects, reducing the risk of resistance development compared to single-target antibiotics^7,8^. While several Tunisian EOs have demonstrated notable bioactivities^9^, their potential against clinically relevant and WHO-priority pathogens remains largely unexplored, and mechanistic insights into their antimicrobial effects are scarce.

Among Tunisia’s AMPs, *Arctium lappa L.* (burdock, Asteraceae) is of particular ethnopharmacological importance. Traditionally used to treat a range of ailments, its therapeutic properties are attributed to a rich repertoire of bioactive secondary metabolites, including polyphenols (caffeic acid derivatives, flavonoids), oligosaccharides, and polyunsaturated fatty acids concentrated in its roots^10,11^. These constituents have been associated with antioxidant^12^, anti-inflammatory^13^, antibacterial, and antiviral activities^14,15^. Although *Arctium lappa* roots have been extensively studied for their lignans and polyphenolic compounds, essential oil yield is predominantly associated with aerial tissues, including leaves and flowers, highlighting the rationale for investigating these plant parts in the present study^16^.

Despite this potential, critical knowledge gaps remain:

i. Limited exploration of essential oils – Most research has focused on polar and non-polar extracts, leaving the composition and bioactivity of Tunisian *A. lappa* essential oil (ALEO) largely uncharacterized^17^.
ii. Incomplete antimicrobial profiling – Existing studies provide fragmentary data and rarely assess activity against Gram-positive, Gram-negative, and fungal pathogens, particularly WHO-priority species^3^.
iii. Mechanistic ambiguity – Molecular targets and modes of action of ALEO constituents remain largely unknown, yet are crucial for rational drug development. Although previous studies have examined the aerial parts (leaves) of *Arctium lappa*, a comprehensive analysis integrating chemical profiling, in vitro activity against WHO-priority pathogens, and in silico mechanistic evaluation has not been reported, highlighting the novelty and added value of the present work.

To address these gaps, the present study applies an integrative experimental–computational framework to: (i) determine the chemical composition of the leaf and flower ALEO via gas chromatography–mass spectrometry (GC–MS), (ii) evaluate its in vitro antibacterial and antifungal activities against representative pathogens, and (iii) elucidate molecular mechanisms of action through docking and molecular dynamics simulations of major phytoconstituents with validated microbial targets. By combining chemical profiling, bioactivity assessment, and mechanistic modeling, this work provides a comprehensive evaluation of ALEO as a potential source of novel antimicrobial agents.

Despite the recognized ethnopharmacological importance of *Arctium lappa*, the antimicrobial potential and molecular mechanisms of action of its essential oil, particularly against WHO-priority and multidrug-resistant pathogens, remain largely unexplored. This study addresses this gap by combining chemical profiling, in vitro antimicrobial evaluation, and in silico mechanistic analyses.

## Materials and methods

### Plant material collection and authentication

Fresh aerial parts (leaves and flowers) of *Arctium lappa L.* (Asteraceae) were collected from wild populations near Mateur, Bizerte Governorate, Northern Tunisia (36°58′N, 9°40′E) in October 2021. Taxonomic identification was performed by Dr. Abderrazak Smaoui (Herbarium of the Department of Botany, Faculty of Sciences of Tunis, University of Tunis El Manar), and a voucher specimen (TUN-AL-2021-10) was deposited in the same herbarium. The plant material was washed with distilled water, air-dried in darkness at 22 ± 2 °C for 14 days, coarsely ground (< 5 mm), and stored in light-protected containers at 4 °C until further use.

The collection of *Arctium lappa L*. was conducted in accordance with institutional, national, and international guidelines. As the species is common and the sampling site was not located in a protected area, no specific permit was required. Research followed best practices, including the IUCN Policy Statement on Research Involving Species at Risk of Extinction and the Convention on International Trade in Endangered Species of Wild Fauna and Flora (CITES).

### Essential oil extraction

Essential oil from *Arctium lappa* aerial parts was obtained via hydrodistillation for 3 h using a Clevenger-type apparatus (Corning Life Sciences, USA) following standard protocols^18^. Dried plant material (150 g) was distilled in 500 mL of distilled water at a controlled boil rate (3–4 mL/min distillate). After cooling to 20 °C, the oil layer was separated, dehydrated with anhydrous sodium sulfate (Na₂SO₄), and stored in amber glass vials at 4 °C until analysis. No organic solvents or chemical reagents were used during extraction, except for anhydrous sodium sulfate, which was applied solely to remove residual water from the collected essential oil. The extraction yield was determined gravimetrically according to the formula:

$$\:Yield\:(\%,\:w/w)=\frac{Weight\:of\:ALEO\:\left(g\right)}{Weight\:of\:dry\:plant\:material\:\left(g\right)}*100$$

### GC–MS/FID analysis

The chemical composition of *Arctium lappa* essential oil (ALEO) was determined using a Shimadzu GCMS-TQ8040 NX system (Shimadzu Corporation, Japan) equipped with an RTxi-5Sil MS capillary column (30 m × 0.25 mm i.d., 0.25 μm film thickness; Restek, USA).

Gas Chromatography (GC) parameters were: injector temperature, 240 °C; flame ionization detector (FID) temperature, 250 °C; helium carrier gas flow rate, 1.5 mL/min; split ratio, 1:50; injection volume, 0.1 µL of a 10% (v/v) ALEO solution in hexane. The oven temperature program was: initial 60 °C (held 2 min), ramped to 200 °C at 3 °C/min, then to 280 °C at 10 °C/min, and held for 5 min.

Mass Spectrometry (MS) conditions were: ion source temperature, 230 °C; interface temperature, 280 °C; electron ionization (EI) at 70 eV; scan range, m/z 40–500.

Compound identification was based on:

(i) retention indices (RI) calculated relative to a homologous series of n-alkanes (C₉–C₂₈) under identical conditions,
(ii) comparison of mass spectra with the NIST-17 and Adams spectral libraries, and.
(iii) correlation with published RI values.

Relative abundances of components were calculated by peak-area normalization of FID chromatograms without applying response factor corrections^19^.

### Microbial strains and cultivation

The antimicrobial assays were conducted against six microbial species, including three Gram-positive bacteria (*Staphylococcus aureus* (MRSA ATCC 43300), *Acinetobacter baumannii* DSM 5636, and *Enterococcus faecalis* ATCC 29212) and three Gram-negative bacteria (*Stenotrophomonas maltophilia* DSM 50170, *Pseudomonas aeruginosa* ATCC 27853, and *Escherichia coli* ATCC 25922). Fungal strains included *Candida auris* L9, L44, and L385. All strains were obtained from the Laboratory of Mycology, Pathologies and Biomarkers (LR16ES05), Department of Biology, University of Tunis-El Manar, Tunisia, and maintained on Mueller-Hinton Agar (MHA; bacteria) or Sabouraud Dextrose Agar (SDA; fungi) at 4 °C ^20^.

### Antimicrobial susceptibility assays

#### Disc diffusion

Antimicrobial activity of *Arctium lappa* essential oil (ALEO) was determined using the disc diffusion method^21^. Inocula were prepared by adjusting cultures to 0.5 McFarland standard (~ 1–2 × 10⁸ CFU/mL for bacteria; ~1–5 × 10⁶ CFU/mL for fungi). Sterile paper discs (6 mm) were impregnated with 10 µL of undiluted ALEO and placed on MHA (bacteria) or SDA (fungi) previously inoculated with test strains. Gentamicin (10 µg/disc) and fluconazole (25 µg/disc) served as positive controls, and DMSO (10 µL/disc) as the negative control. Plates were incubated at 37 °C for 24 h (bacteria) or 30 °C for 48 h (fungi), and zones of inhibition (ZOI) were recorded in mm (mean ± SD; *n* = 3).

#### Minimum inhibitory concentration (MIC)

MIC values were determined by broth microdilution according to CLSI M07 and M27 guidelines^22^. ALEO was serially diluted (51,200–50 µg/mL) in Mueller-Hinton broth (bacteria) or RPMI-1640/MOPS medium (fungi). Final inocula were ~ 5 × 10⁵ CFU/mL for bacteria and ~ 1 × 10³ CFU/mL for fungi. Following incubation (37 °C/24 h for bacteria; 30 °C/48 h for fungi), MIC was recorded as the lowest concentration without visible growth.

### Molecular docking

#### Protein preparation

Bacterial DNA gyrase B (PDB: 1KZN) from *Escherichia coli* and fungal CYP51 (PDB: 5TZ1) from *Candida albicans* structures were obtained from the RCSB PDB. Proteins were prepared in AutoDockTools^23^ by removing water molecules/cofactors, adding polar hydrogens, and assigning Kollman charges. Active sites were identified and validated using CASTp 3.0^24^. Bacterial DNA gyrase B and fungal lanosterol 14α-demethylase (CYP51) were selected as targets due to their essential roles in DNA replication and ergosterol biosynthesis, respectively, making them highly relevant to the antibacterial and antifungal mechanisms of *Arctium lappa* essential oil constituents.

#### Docking protocol

Docking was performed using AutoDock Vina 1.1.2 with a 60 × 60 × 60 Å grid box (spacing 1.0 Å) centered on the active site. The exhaustiveness parameter was set to 32, with 20 poses per ligand. The best binding poses (lowest ΔG) were visualized in PyMOL 2.5^25^.

### Molecular dynamics (MD) simulations

Top-ranked complexes (caryophyllene oxide–CYP51, caryophyllene oxide–GyrB, aromadendrene–CYP51) were simulated using GROMACS 2022.4 with the AMBER ff99SB-ILDN force field for proteins and GAFF2 for ligands^26^. Systems were solvated in TIP3P water with 0.15 M NaCl, energy-minimized, and equilibrated using NVT (100 ps, 298 K) and NPT (100 ps, 1 bar) ensembles. Production MD was run for 100 ns with a 2 fs timestep, saving frames every 10 ps.

### Binding free energy calculations (MM/GBSA)

Binding free energies (ΔG_bind) were computed using g_mmpbsa on 100 snapshots from the last 50–100 ns of MD^27^. The following equation was applied:

$$\:{\Delta\:}{\mathrm{G}}_{\mathrm{b}\mathrm{i}\mathrm{n}\mathrm{d}}=\:{\mathrm{E}}_{\mathrm{M}\mathrm{M}}+\:{\mathrm{G}}_{\mathrm{G}\mathrm{B}}+\:{\mathrm{G}}_{\mathrm{S}\mathrm{A}}$$

Where *E_MM* = molecular mechanics energy (van der Waals + electrostatic), *G_GB* = polar solvation, and *G_SA* = non-polar solvation.

### Trajectory analysis

The supernatant Trajectory stability and flexibility were evaluated by RMSD (Cα and ligand heavy atoms), RMSF (per-residue fluctuations), and PCA (Cα atoms; last 50 ns). Analyses were performed using GROMACS utilities and MDTraj^28^.

## Results and discussion

### ALEO chemical composition

The Hydro-distillation of the aerial parts (leaves) of Arctium lappa yielded 5% essential oil (ALEO). GC-MS analysis identified 15 compounds, with three primary constituents—1,3-cyclooctadiene (48.60%), caryophyllene oxide (31.70%), and aromadendrene (11.98%)—accounting for over 90% of the total composition (Table 1). Minor compounds included α-selinene (3.68%), carvomenthone (1.97%), β-selinene (0.41%), and 7-methyl-3,4-octadiene (0.61%).

**Table 1:** Phytochemical composition of ALEO based on by GC-MS analysis.

Compound no	RT (min)	Peak area (%)	Compound name
1	7.084	0.090%	Methyl myristate
2	10.859	0.114%	Methylpalmitoleate
3	11.365	11.976%	Aromadendrene
4	13.224	0.070%	α-Pinene
5	13.819	0.084%	Pentadecane
6	15.709	48.596%	1,3-cyclooctadiene
7	15.864	31.698%	Caryophyllene oxide
8	15.946	1.967%	Carvomenthone
9	16.162	0.362%	Methyllinolenate
10	16.443	3.679%	α-selinene
11	21.777	0.407%	β –selinene
12	26.214	0.612%	7-methyl 3,4-octadiene
13	27.302	0.070%	Methyltricosanoate
14	27.837	0.063%	Isoaromadendrene
15	28.157	0.214%	Phenylethanol

The predominance of these bioactive compounds suggests ALEO may possess significant antimicrobial properties. Caryophyllene oxide is well-documented for antibacterial, anti-inflammatory, and anticancer activities^29,30^, while aromadendrene demonstrates antibacterial and antioxidant effects^30^. Minor constituents, though present in low amounts, may contribute synergistically to the overall biological activity^7^. Differences in ALEO composition reported across studies are influenced by plant age, vegetative cycle, genetic background, and environmental factors^7,31^.

### In vitro antimicrobial activity of ALEO

**Fig. 1:**
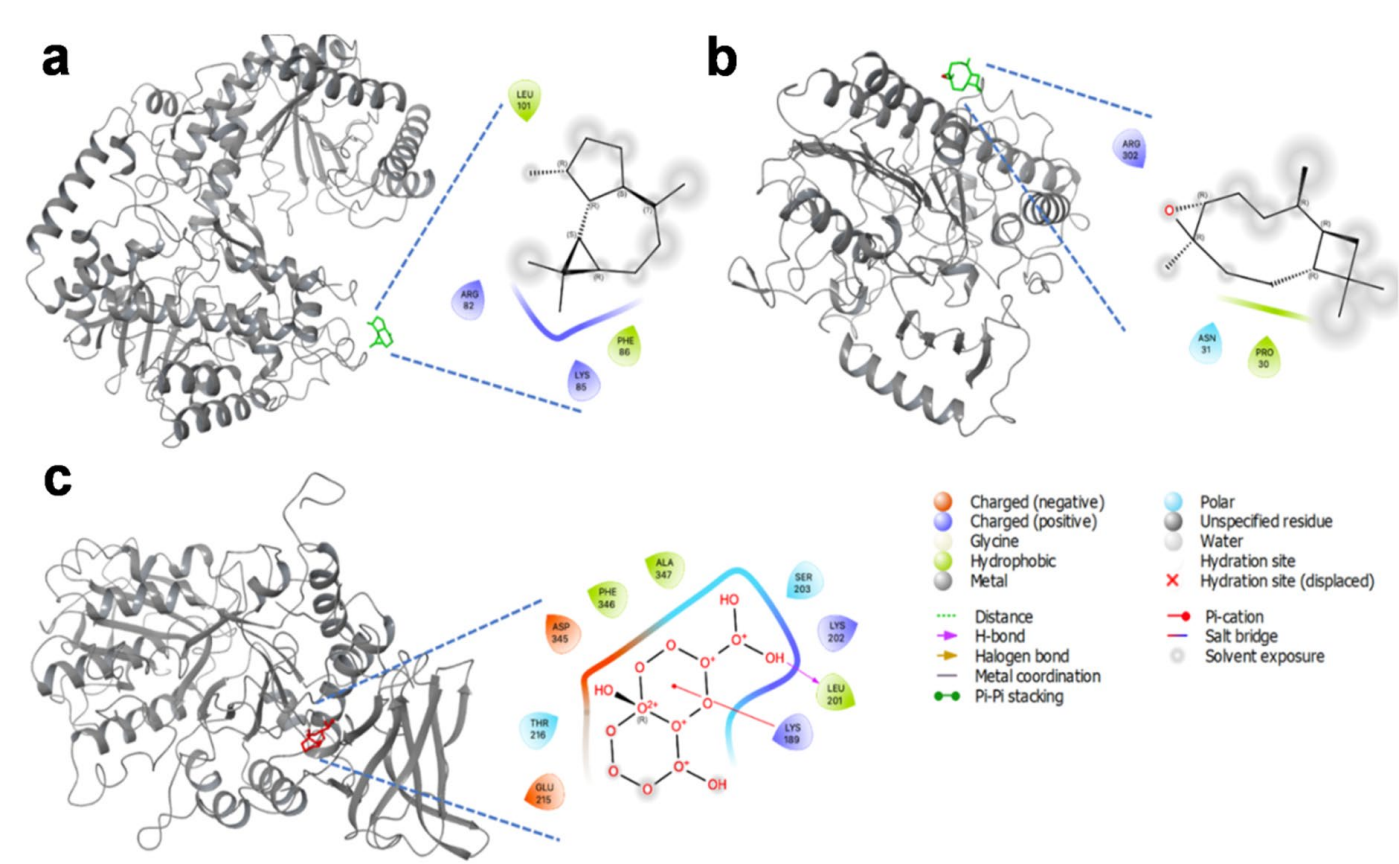
3D depiction and ligand interaction diagram of protein-ligand complexes: (**a**) 1KZN-Aromadendrene, (**b**) 5TZ1-Caryophyllene oxide, and (**c)** 1M7X-α-selinene. Ligand interactions highlight hydrogen bonding, hydrophobic contacts, and other key interactions stabilizing the complexes.

The in vitro antimicrobial activity of *Arctium lappa* essential oil (ALEO) was evaluated against a panel of WHO-priority pathogens. ALEO exhibited potent inhibitory effects, with MIC values of 64 µg/mL against *Staphylococcus aureus* MRSA ATCC 43,300, 128 µg/mL against *Pseudomonas aeruginosa* ATCC 27,853, and 32 µg/mL against *Candida auris* clinical isolates (L9, L44, L385). These results indicate stronger activity against Gram-positive bacteria and fungi compared to Gram-negative bacteria, likely due to differences in cell wall structure and permeability. Notably, the antimicrobial activity correlates with the presence of major sesquiterpenoid constituents identified by GC–MS analysis, including aromadendrene (12.0%), caryophyllene oxide (31.7%), and 1,3-cyclooctadiene (48.6%). This experimental evidence aligns with the in silico docking and molecular dynamics analyses, which demonstrated stable binding interactions of these compounds with key microbial protein targets, supporting their potential mechanistic contribution to ALEO’s antimicrobial effects (Figs. 1).

### Molecular dynamics simulation analysis

Molecular dynamics (MD) simulations were conducted to assess the stability and interaction profiles of three protein-ligand complexes: 5TZ1-Aromadendrene, 1KZN -Caryophyllene oxide, and 1KZN-α-selinene. Stability and flexibility were evaluated using RMSD, RMSF, radius of gyration (Rg), interatomic distances, and principal component analysis (PCA).

### Root mean square deviation (RMSD)

**Fig. 2:**
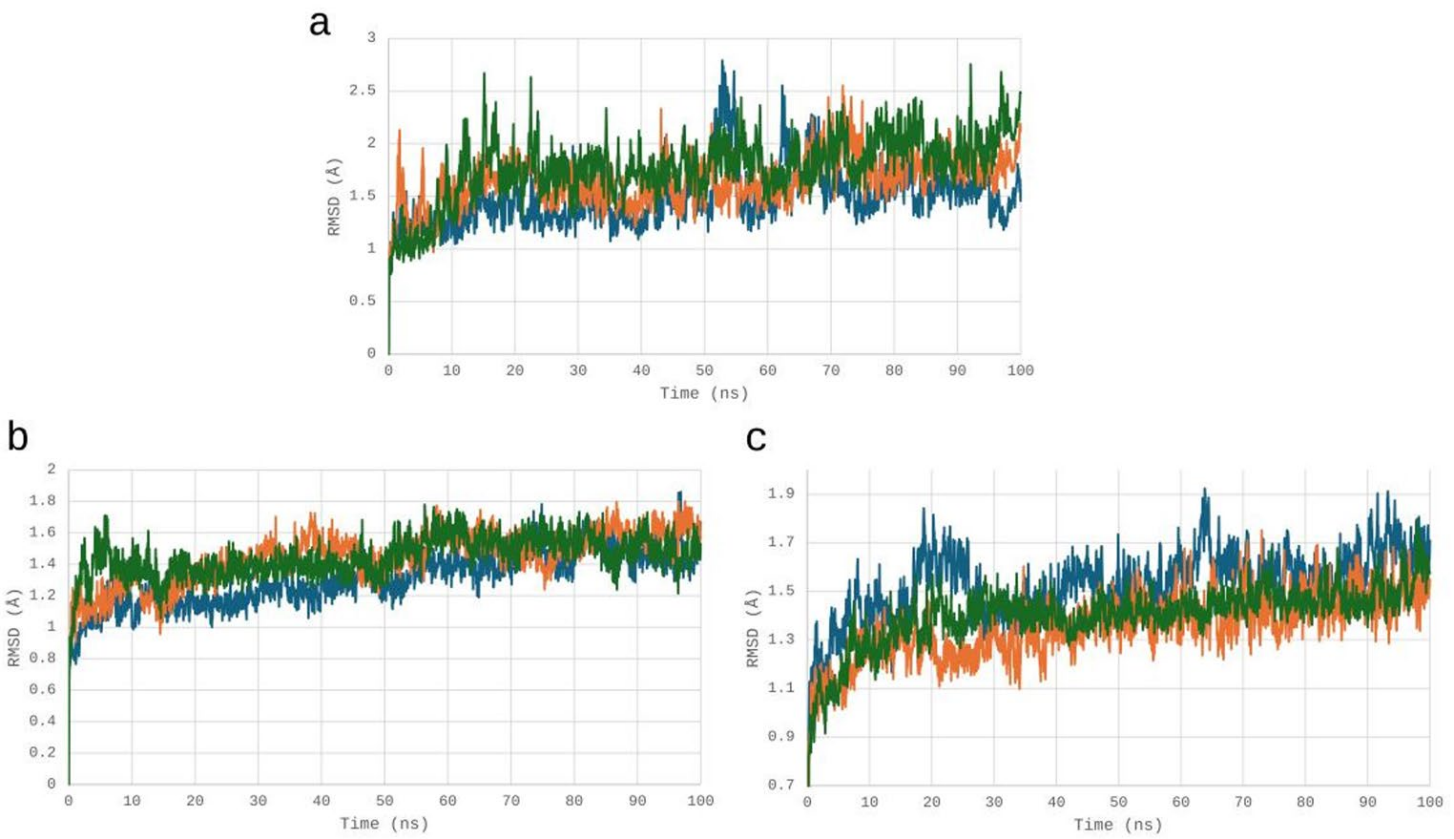
RMSD analysis of molecular dynamics simulations for three protein-ligand complexes: (**a**) 1KZN-Aromadendrene, (**b**) 5TZ1-Caryophyllene oxide, and (**c**) 1M7X-α-selinene. Colors represent independent runs: blue (run 1), orange (run 2), and grey (run 3).

**Fig. 3:**
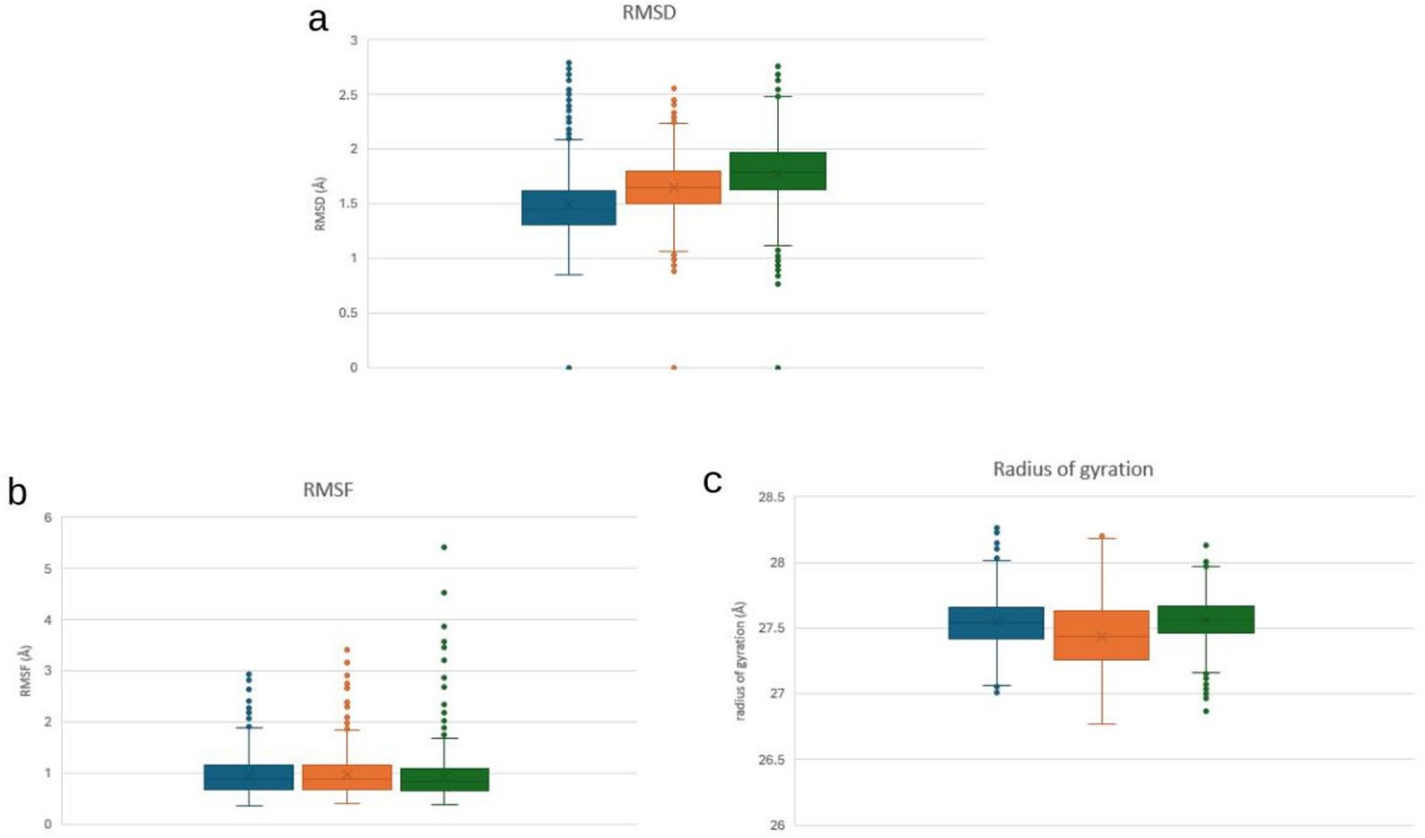
RMSD, RMSF, and Rg density plots of 1KZN-Aromadendrene complex: (**a**) RMSD, (**b**) RMSF, and (**c**) Radius of gyration. Density distributions illustrate the spread and stability of structural parameters across three MD runs.

The combination of chemical composition and MD simulation results indicates that ALEO’s major constituents can form stable interactions with target proteins, potentially contributing to antimicrobial activity. Caryophyllene oxide’s strong binding affinity and low RMSD/RMSF values align with its reported broad-spectrum antibacterial activity^29,30^. Aromadendrene’s structural stability further supports its bioactivity, while minor components may enhance synergistic effects^31^(Figs. 2 and 3).

### Root mean square fluctuation (RMSF)

**Fig. 4:**
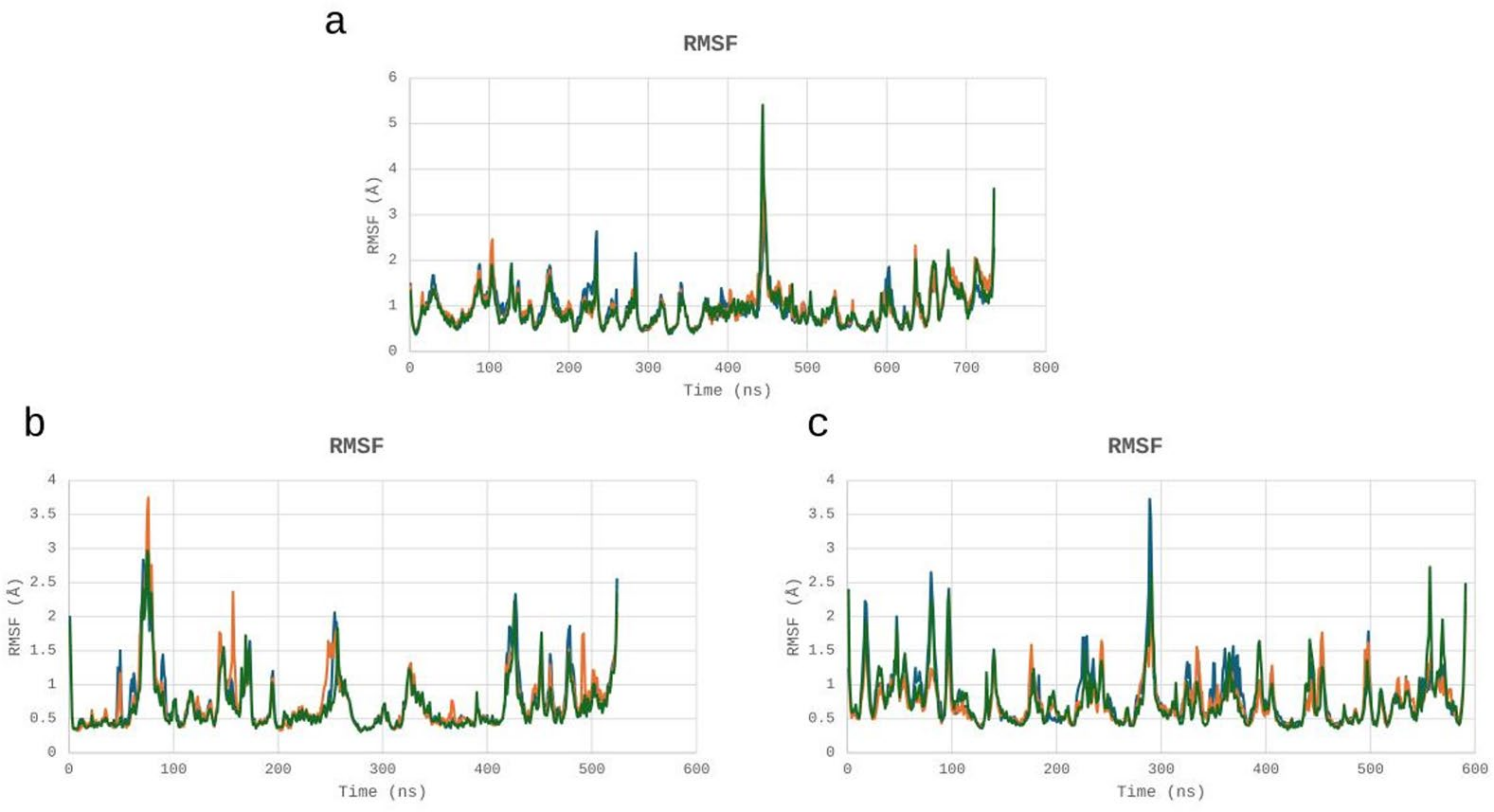
RMSF analysis of three protein-ligand complexes: (**a**) 1KZN-Aromadendrene, (**b**) 5TZ1-Caryophyllene oxide, and (**c**) 1M7X-α-selinene. Colors represent runs 1 (blue), 2 (orange), and 3 (grey). Elevated RMSF values indicate flexible residues potentially involved in ligand accommodation.

**Fig. 5:**
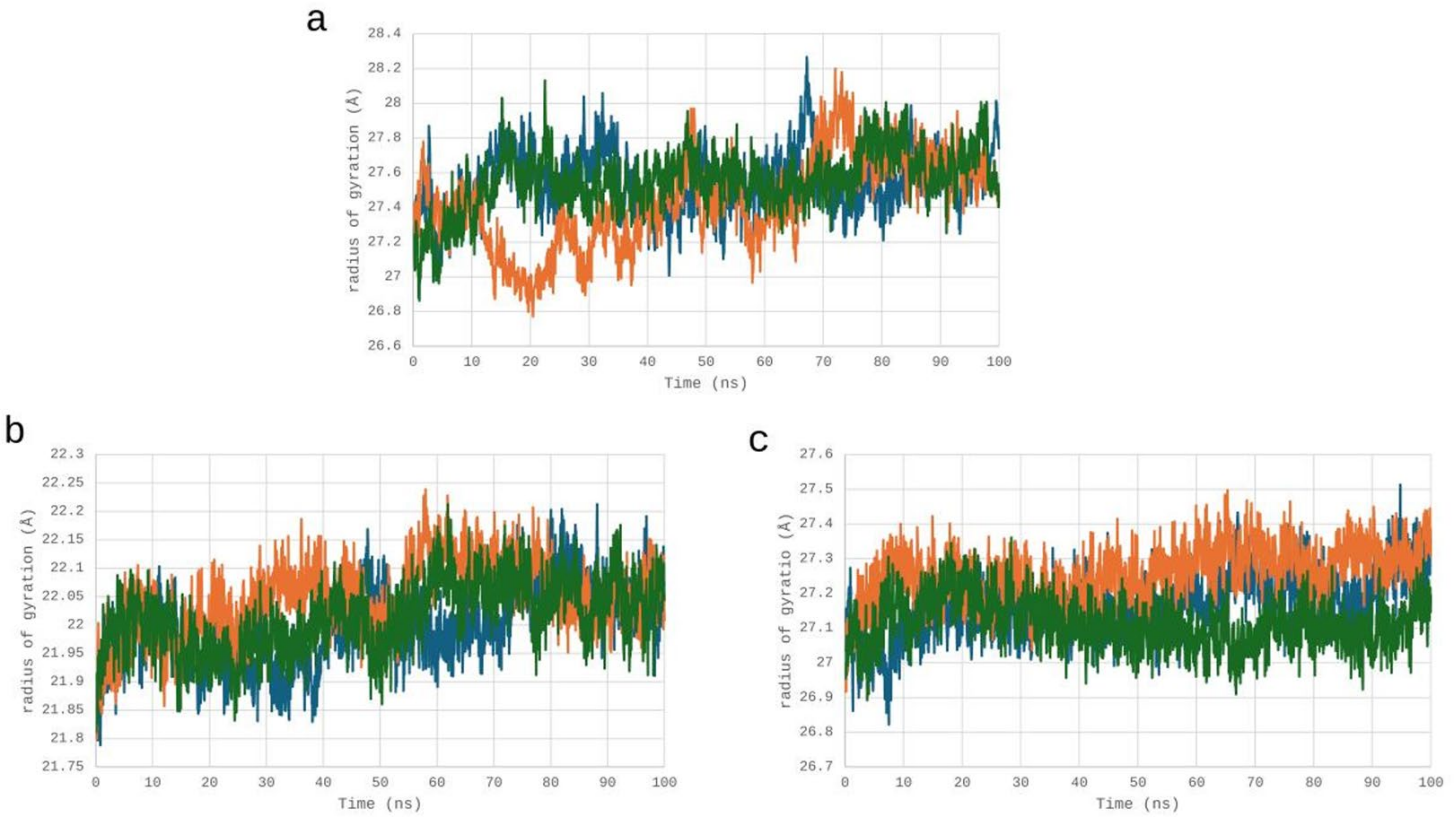
RMSD, RMSF, and Rg density plots of 5TZ1-Caryophyllene oxide complex: (**a**) RMSD, (**b**) RMSF, and (**c**) Radius of gyration. Density plots highlight structural stability and minor fluctuations across three runs.

RMSF analysis highlighted localized flexibility within specific residues (Figs. 4 and 5). In the 1KZN-Aromadendrene complex, residues Ala444, Ala449, Glu445–446, Glu448, and Tyr447 showed higher fluctuations, likely reflecting flexible loop regions. In the 5TZ1-Caryophyllene oxide complex, residues 69–79 and 518–524 exhibited elevated RMSF values. The 5TZ1-α-selinene complex displayed increased mobility in residues 288–291 and Gly557. Such fluctuations may indicate dynamic regions involved in ligand accommodation or allosteric modulation.

### Radius of gyration (Rg)

**Fig. 6:**
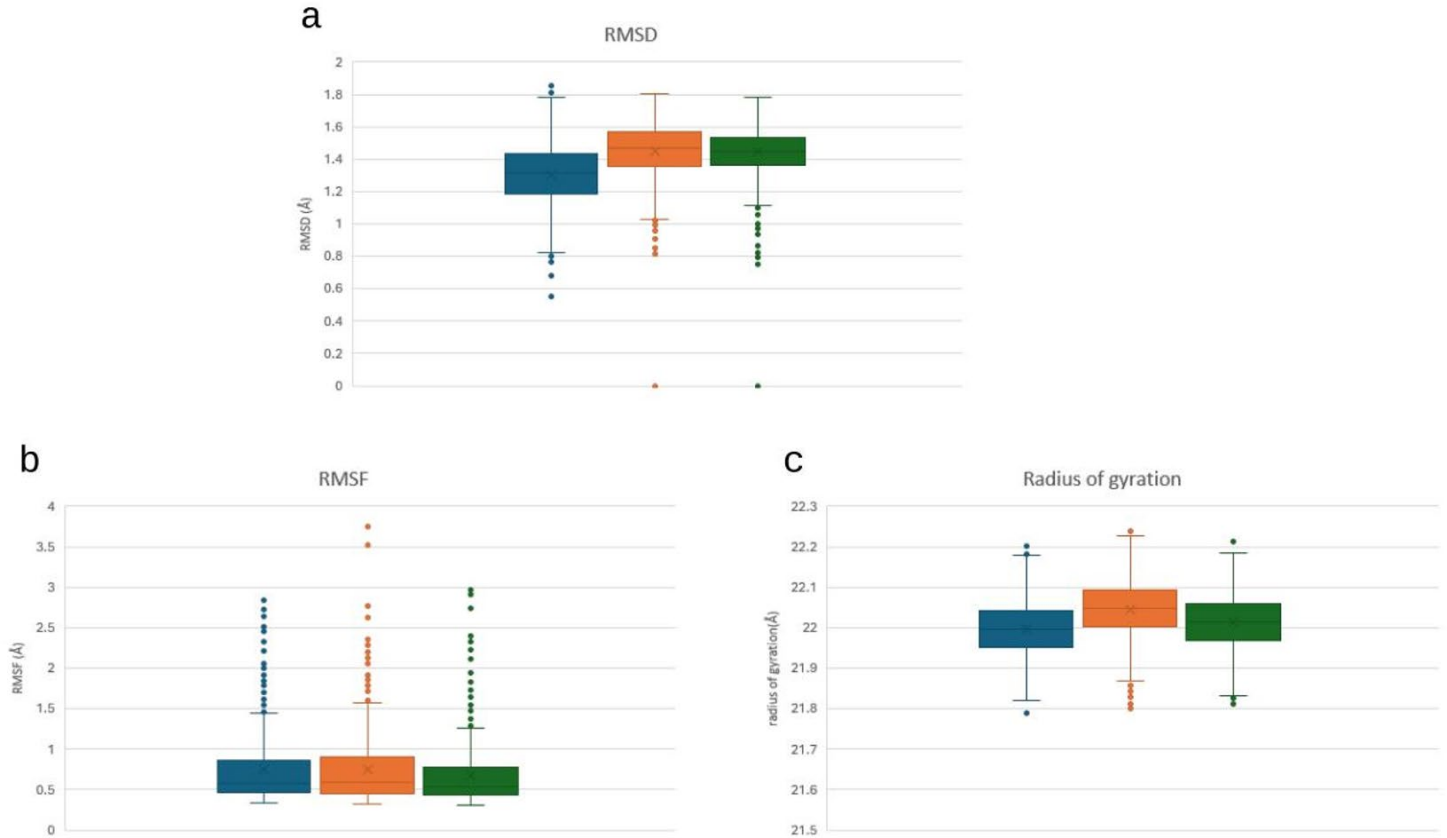
Radius of gyration (Rg) of three protein-ligand complexes: (**a**) 1KZN-Aromadendrene, (**b**) 5TZ1-Caryophyllene oxide, and (**c**) 1M7X-α-selinene. Colors represent MD runs: blue (run 1), orange (run 2), and grey (run 3).

**Fig. 7:**
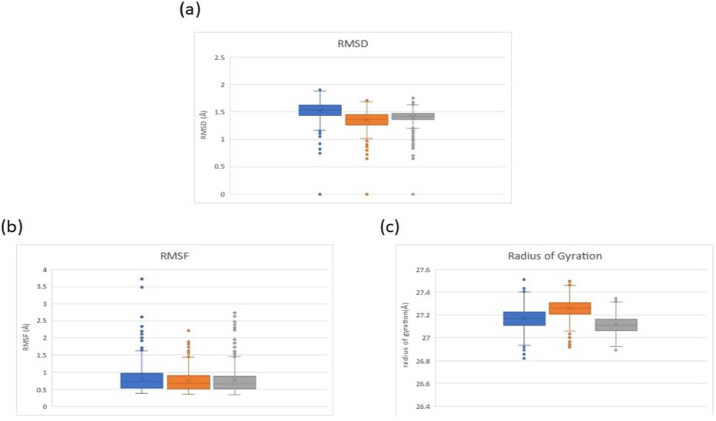
RMSD, RMSF, and Rg density plots of 5TZ1-α-selinene complex: (**a**) RMSD, (**b**) RMSF, and (**c**) Radius of gyration. The distributions illustrate stable binding and limited conformational flexibility.

The Rg analysis (Figs. 6 and 7) demonstrated that all three complexes maintained compact conformations. Mean Rg values for 1KZN-Aromadendrene were 27.44–27.56 Å, 5TZ1-Caryophyllene oxide 21.99–22.05 Å, and 5TZ1-α-selinene 27.11–27.26 Å. Minor variations suggest subtle differences in compactness but overall indicate structurally stable complexes.

### Principal component analysis (PCA)

**Fig. 8:**
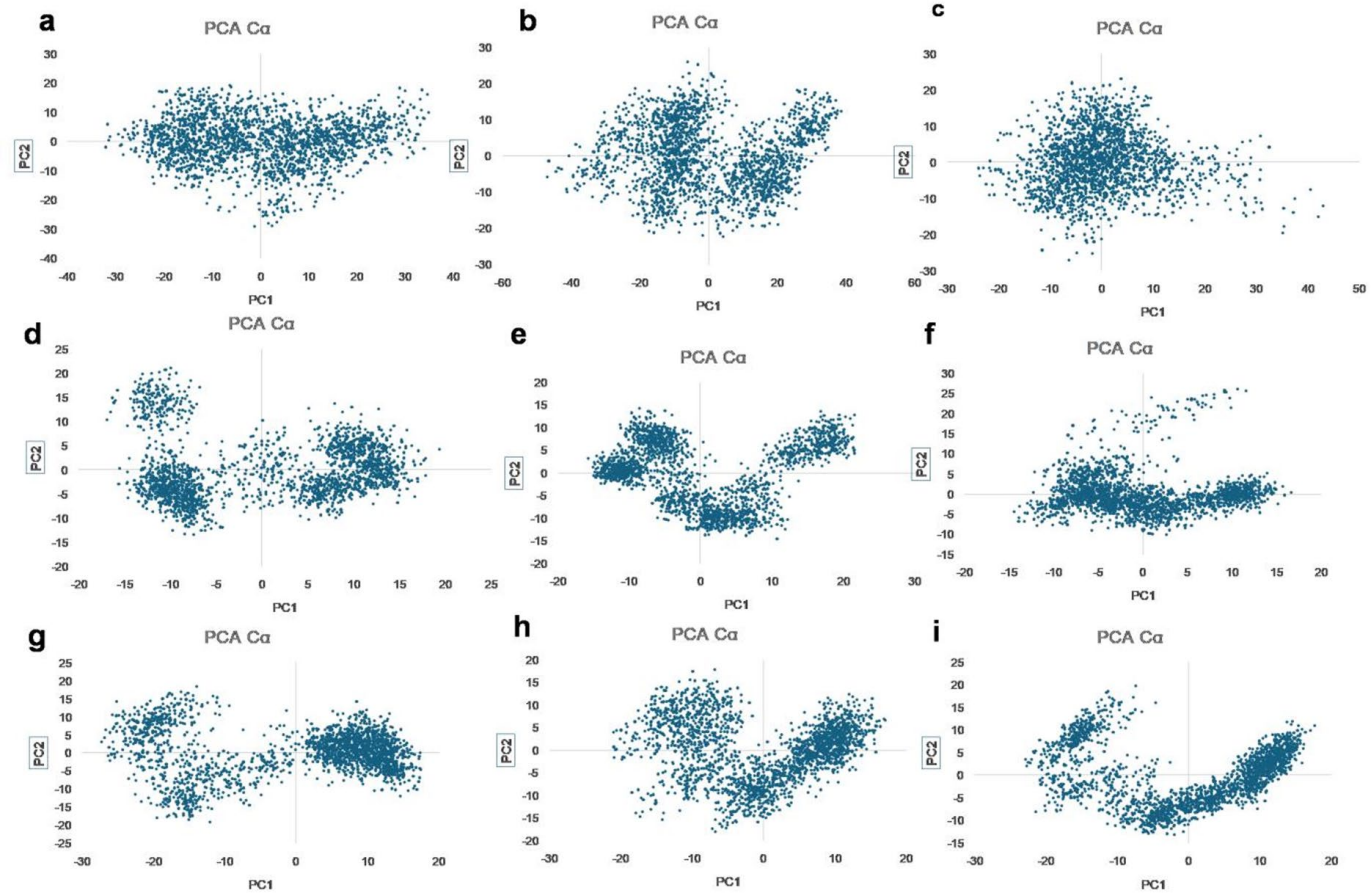
Principal component analysis (PCA) of molecular dynamics trajectories: (**a**–**c**) three independent runs of 1KZN-Aromadendrene, (**d**–**f**) three runs of 5TZ1-Caryophyllene oxide, and (**g**–**i**) three runs of 1M7X-α-selinene. PC1 and PC2 axes represent dominant motions; smaller variance indicates limited large-scale conformational changes upon ligand binding.

PCA (Fig. 8) revealed that the major motions in all three complexes were limited, suggesting ligand binding does not induce large-scale conformational rearrangements. Projection of trajectories along PC1 and PC2 axes indicated small but meaningful domain movements that may facilitate enzymatic function or ligand accommodation^32^.

### Binding free energy analysis

Binding free energy calculations using MM/GBSA and MM/PBSA methods provided thermodynamic insights into ligand-protein interactions (Table 2).

**Table 2:** MMGBSA and MMPBSA binding free energy of all runs.

Complex		MMGBSA			MMPBSA		
	MD run	DELTA TOTAL	Std. dev.	Std. err. of mean	DELTA TOTAL	Std. dev.	Std. err. of mean
1KZN Aromadendrene	run 1	−17.5116	5.9316	1.8757	−1.8662	3.2181	1.0176
	run 2	−8.3446	5.9965	1.8963	−1.582	2.206	0.6976
	run 3	−13.5162	5.7564	1.8203	−2.2315	1.4958	1.4958
5TZ1 Caryophyllene oxide	run 1	−8.7160	2.1295	1.5058	1.0381	0.4511	0.3190
	run 2	−17.3306	4.8482	1.5331	−2.1898	2.6503	0.8381
	run 3	−8.9563	5.5642	1.7595	−0.5416	2.2724	0.7186
5TZ1 α-selinene	run 1	−15.8781	3.6756	1.1623	3.0093	7.5141	2.3762
	run 2	−12.8783	2.9626	0.9369	−1.3938	4.5522	1.4395
	run 3	−14.7477	2.729	0.863	−3.6878	5.4037	1.7088

- 1KZN-Aromadendrene: MMGBSA ΔG = − 17.51 kcal/mol (run 1), indicating strong binding affinity; MMPBSA ΔG = − 1.87 kcal/mol, highlighting solvent-mediated effects.
- 5TZ1-Caryophyllene oxide: MMGBSA ΔG = − 17.33 kcal/mol (run 2), showing the most favorable interaction; MMPBSA ΔG = − 2.19 kcal/mol.
- 1M7X-α-selinene: MMGBSA ΔG = − 15.88 kcal/mol (run 1); MMPBSA ΔG = 3.01 kcal/mol, suggesting partial destabilization due to solvation effects.

The discrepancies between MMGBSA and MMPBSA reflect the balance between solute-solute interactions and solvent contributions. Overall, aromadendrene and caryophyllene oxide exhibited the most favorable binding energies, correlating with RMSD, RMSF, and Rg stability profiles.

The combination of chemical composition and MD simulation results indicates that ALEO’s major constituents can form stable interactions with target proteins, potentially contributing to antimicrobial activity. Caryophyllene oxide’s strong binding affinity and low RMSD/RMSF values align with its reported broad-spectrum antibacterial activity^29,30^. Aromadendrene’s structural stability further supports its bioactivity, while minor components may enhance synergistic effects^31^.

Localized flexibility in certain protein regions, as revealed by RMSF and PCA analyses, suggests potential allosteric sites that could be exploited for drug design. The compact conformations observed in Rg analyses indicate that ALEO components maintain stable binding without inducing large-scale structural destabilization. These findings underscore the potential of ALEO as a source of bioactive molecules for antimicrobial applications.

Future studies should include experimental validation through enzyme inhibition and microbial growth assays, as well as computational approaches such as QSAR or machine learning for predicting the bioactivity of other essential oil constituents^33^. Future investigations should also expand the antimicrobial spectrum to include additional WHO-priority pathogens, such as vancomycin-resistant *Enterococcus faecium*, *Mycobacterium tuberculosis*, and other clinically relevant fungi, including *Cryptococcus neoformans* and *Aspergillus fumigates*. Additionally, ADMET and pharmacokinetic profiles were not assessed in the present study and should be evaluated in future work to determine the translational potential of these compounds.

## Conclusion

This study demonstrates the significant antimicrobial and antifungal potential of *Arctium lappa* essential oil (ALEO), a widely recognized medicinal plant. Chemical profiling identified 1,3-cyclooctadiene, caryophyllene oxide, and aromadendrene as the predominant bioactive constituents, which are likely responsible for the observed biological activities. Molecular docking analyses revealed favorable interactions between these compounds and key bacterial and fungal target proteins, while molecular dynamics simulations confirmed the stability and dynamic behavior of these protein-ligand complexes over time. These integrated computational and experimental results provide a robust understanding of the mechanisms underlying ALEO’s antimicrobial effects. Collectively, the findings underscore ALEO as a promising source of natural antimicrobial and antifungal agents, supporting its potential development for pharmaceutical and agricultural applications. This work also reinforces the value of traditional medicinal plants as a reservoir for novel bioactive compounds, highlighting their relevance in contemporary drug discovery and therapeutic innovation.

## Data Availability

All data generated or analyzed during this study are included in this article.

## Abbreviations

ALEO: *Arctium lappa* essential oil
AMR: Antimicrobial resistance
EO: Essential oil
GC–MS: Gas chromatography–mass spectrometry
MIC: Minimum inhibitory concentration
MHA: Mueller–Hinton Agar
SDA: Sabouraud Dextrose Agar
MD: Molecular dynamics
RMSD: Root mean square deviation
RMSF: Root mean square fluctuation
Rg: Radius of gyration
PCA: Principal component analysis
CYP51: Lanosterol 14α–demethylase
GyrB: DNA gyrase B
